# Difference in the distribution pattern of substrate enzymes in the metabolic network of *Escherichia coli*, according to chaperonin requirement

**DOI:** 10.1186/1752-0509-5-98

**Published:** 2011-06-24

**Authors:** Kazuhiro Takemoto, Tatsuya Niwa, Hideki Taguchi

**Affiliations:** 1 PRESTO, Japan Science and Technology Agency, Kawaguchi, Saitama 332-0012, Japan; 2 Department of Biophysics and Biochemistry, University of Tokyo, Hongo 7-3-1, Bunkyo-ku, Tokyo 113-0033, Japan; 3 Department of Biomolecular Engineering, Tokyo Institute of Technology, Nagatsuta 4259-B56, Midori-ku, Yokohama 226-8501, Japan

## Abstract

**Background:**

Chaperonins are important in living systems because they play a role in the folding of proteins. Earlier comprehensive analyses identified substrate proteins for which folding requires the chaperonin GroEL/GroES (GroE) in *Escherichia coli*, and they revealed that many chaperonin substrates are metabolic enzymes. This result implies the importance of chaperonins in metabolism. However, the relationship between chaperonins and metabolism is still unclear.

**Results:**

We investigated the distribution of chaperonin substrate enzymes in the metabolic network using network analysis techniques as a first step towards revealing this relationship, and found that as chaperonin requirement increases, substrate enzymes are more laterally distributed in the metabolic. In addition, comparative genome analysis showed that the chaperonin-dependent substrates were less conserved, suggesting that these substrates were acquired later on in evolutionary history.

**Conclusions:**

This result implies the expansion of metabolic networks due to this chaperonin, and it supports the existing hypothesis of acceleration of evolution by chaperonins. The distribution of chaperonin substrate enzymes in the metabolic network is inexplicable because it does not seem to be associated with individual protein features such as protein abundance, which has been observed characteristically in chaperonin substrates in previous works. However, it becomes clear by considering this expansion process due to chaperonin. This finding provides new insights into metabolic evolution and the roles of chaperonins in living systems.

## Background

Understanding metabolic activities in the body is important because metabolism is responsible for physiological functions and thus maintaining life. Metabolism can be defined as a series of chemical reactions involving enzymes as catalysts, and these reactions are often represented as a network (called metabolic networks) [1-3]. In recent years, considerable genomic data and metabolic network data have been accumulated using several new technologies and high-throughput methods. Thus, research on this topic was actively carried out with comprehensive analyses of metabolic networks, and the entire picture of metabolic networks steadily became clearer (reviewed in [4,5]). Here, we have discussed the mechanisms involved in the evolution of metabolic networks [6-8] and the environmental adaptation from the viewpoint of metabolism [9-12].

Protein folding is an important aspect of metabolism because normal metabolism requires proper functioning of cellular enzymes (i.e., the satisfactory conformation and function of native enzyme structures). According to Anfinsen's dogma [13], the unique native structure of a protein is determined by its amino acid sequence. Although proteins are usually present in their native form, according to this dogma, they often aggregate due to environmental stress and other factors.

Chaperones, most of which are heat-shock proteins, assist in protein folding, and they prevent the misfolding and aggregation of proteins (reviewed in [14,15]). In particular, in *Escherichia coli*, the chaperonin GroEL, together with its cofactor GroES, acts a chaperone system, which assists in protein folding in this organism, and is essential under several growth conditions (temperatures) [16]. The indispensability of chaperonins is also suggested by the observation that many proteins tend to aggregate in chaperonin-free cells of *E. coli *[17]. Therefore, it is important to determine the role of the chaperonin GroEL/GroES (GroE) in living systems.

Until now, several GroE substrates have been identified. As a conclusive method for identifying chaperonin substrates, a detailed analysis of the phenotypes of GroE-depleted cells is often utilized [18,19]. This approach can evaluate the exact chaperonin requirement of substrates; however, it has limitations because it is difficult to comprehensively determine chaperonin requirement. On the other hand, an exhaustive proteome-wide analysis has identified chaperonin substrates [20,21]. In particular, Kerner *et al*. [20] identified about 250 chaperonin substrates by using mass spectrometry, and they classified these substrates into several groups according to their chaperonin requirement (see Results and Discussion for details). Furthermore, Fujiwara *et al*. comprehensively reinvestigated chaperonin-dependent substrates on the basis of proteomics, metabolomics, and individual requirements for chaperonin in cells [22], because the previous works did not investigate chaperonin dependence for most of the substrates *in vivo*. As a result, they could more precisely identify obligate chaperonin substrates (see Results and Discussion for details).

These previous works found that many chaperonin substrates correspond to metabolic enzymes [16,20,22]. For example, Fujiwara *et al*. [22] showed that about 70% of obligate chaperonin-dependent substrates are metabolic enzymes. These results indicate the potential importance of chaperonins in metabolism. However, the relationship between chaperonin substrates and metabolism (or the metabolic network) has not been examined until now.

Here, we have investigated the distribution of chaperonin substrates in metabolic networks as a first step towards revealing this relationship, and show 2 main results. The first observation is the nontrivial relationship between the position of substrate enzymes in the network and chaperonin requirement: with the increase in chaperonin requirement, substrate enzymes tend to get more laterally distributed in the metabolic network. The second observation is the lower degree of conservation of chaperonin substrates among organisms, which suggests that chaperonin substrates emerged later on in evolutionary history. From these results, we discuss the origin of the distribution pattern of substrate enzymes in the metabolic network and the roles of chaperonins in the evolution of metabolic networks.

## Results and Discussion

### Survey of the chaperonin substrate classes

We have utilized 2 types of classification schemes to characterize the chaperonin GroE requirement in *E. coli*. We have presented details of the GroE substrate classes because the classification of chaperonin substrates is important for the following data analysis and it is slightly complicated.

Proteins are classified into several groups based on GroE requirement for folding. Kerner *et al*. [20] identified GroE-dependent substrate proteins via proteome-wide analysis, and they classified these substrates into 3 groups: Class I as GroE-independent substrates (i.e., protein folding does not require chaperonin), Class II as partial GroE-dependent substrates (i.e., protein folding depends on chaperonin under certain environmental conditions such as stress), and Class III as potential obligate GroE-dependent substrates (i.e., protein folding requires chaperonin).

However, the previous analysis did not fully confirm the requirement for GroE *in vivo *in folding. Thus, Fujiwara *et al*. [22] investigated the chaperonin-dependent substrates (i.e., Class III) via detailed analysis of the phenotypes of GroE-depleted cells. As a result, the GroE-dependent substrate classes were modified. Fujiwara *et al*. found several novel obligate chaperonin-dependent substrates. Moreover, they revealed that about 60% of Class III substrates require GroE, and that the chaperonin requirements of the remaining (about 40% of Class III) substrates are unclear because these proteins were soluble in the absence of GroE even though they are known to interact with this chaperonin. In addition, they showed that a few Class II substrates are obligate chaperonin-dependent substrates *in vivo*. Therefore, they classified these novel substrates and the subset of Class II and Class III substrates, according to their chaperonin requirements *in vivo*, as Class IV substrates. The 40% of Class III substrates whose chaperonin requirements *in vivo *are unclear were classified as Class III*^- ^*substrates.

We also need to modify the definition for Class II because of a few Class II substrates whose chaperonin dependence was experimentally confirmed. In this paper, we defined Class II' substrates after eliminating Class II substrates requiring GroE *in vivo *from the traditional Class II substrates. However, Class II' is almost similar to Class II because only about 3% of the total Class II substrates were removed.

We have considered 2 classification schemes: Kerner's classification (i.e., Class I, II, and III) and Fujiwara's classification (Class I, II', and IV). In Fijiwara's classification, Class III*^- ^*was omitted because the chaperonin requirement was unclear; however, the difference between Class III*^- ^*and IV has been evaluated in the following section.

### Extraction and classification of metabolic enzymes as chaperonin substrates

Metabolic enzymes were extracted from the whole set of chaperonin substrates explained above because all chaperonin substrates are not metabolic enzymes.

We constructed the metabolic network of *E. coli*, in which the nodes and edges correspond to metabolic reactions (enzymes) and interjacent metabolites, respectively (see Methods for details). Because we used the shortest path analysis in the following section, the metabolic network is represented as a connected network with undirected (and unweighted) edges. The reaction (enzyme) nodes are assigned the corresponding gene identifiers (b-numbers; e.g., b2097 in the case of fructose-bisphosphate aldolase Class I). According to the gene identifier, the metabolic enzymes were divided on the basis of the above 2 classification schemes. In some cases, 1 enzyme has more than 1 gene because it consists of subunits. In this case, counting this enzyme with more than 1 gene belonging to the same chaperonin substrate class was redundant.

The number of enzymes in each substrate class is as follows. With Kerner's classification, we obtained 29 Class I substrate enzymes, 41 Class II substrate enzymes, and 40 Class III substrate enzymes. With Fujiwara's classification, on the other hand, we obtained 29 Class I substrate enzymes (they are similar to Class I of Kerner's classification), 38 Class II' substrate enzymes, and 38 Class IV substrate enzymes. In addition, 9 Class III*^- ^*substrates were observed. In addition, approximately 20% of the enzymes are the chaperonin substrates in the metabolic network.

### Lateral distribution of substrate enzymes in the metabolic network, according to chaperonin requirement

To characterize the relationship between the metabolic network and chaperonin substrate enzymes, we considered the distribution of the substrates in the network. In this section, we focused on the distribution of distance from the center. This feature is characterized by the proportion of substrate enzymes separated by the shortest path length *h *from the central (source) node *o*, and it is defined as follows: , where *d*(*o, x*) is the shortest path length from the source node *o *to node *x*. In addition, *C *is the set of enzymes belonging to its respective substrate class, and *|C| *is the number of elements of the substrate class. *δ*(*x*) is the Kroneker delta function that returns 1 if *x *= 0, and 0 otherwise. We defined the central (source) node as pyruvate kinase for 2 main reasons. Pyruvate is a well-studied and very important metabolite. Many previous works [1-3,23] imply that pyruvate is a central compound in the metabolic network. In fact, pyruvate serves as a connector between many different metabolic pathways such as gluconeogenesis, the citrate cycle, amino acid metabolism, and lipid metabolism. Pyruvate kinase was also considered as the central node because of the gluconeogenic origin of metabolism [24]. Comparative genomic analysis showed that gluconeogenesis is well conserved among wide-ranging species, suggesting that the metabolic pathway started expanding around pyruvate. Although pyruvate kinase is a glycolytic enzyme (not a gluconeogenic one), we decided to make pyruvate kinase the center because it is a well-known enzyme associated with pyruvate, which is believed to be a central compound.

**Figure 1:**
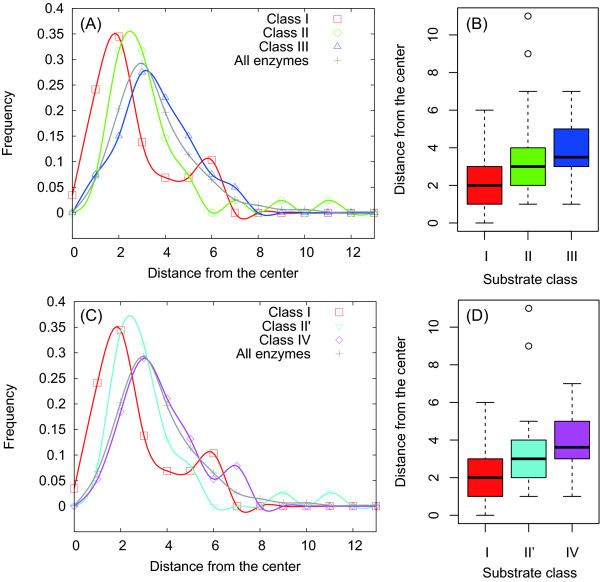
**Distance between chaperonin substrate enzymes and the center**. The distance and center are represented by the shortest path length and pyruvate kinase, respectively. The frequency distributions of the distance according to Kerner's classification (A) and Fujiwara's classification (C). The boxplots of the distance according to Kerner's classification (B) (*P *= 0.009 using the Kruskal-Wallis (KW) test) and Fujiwara's classification (D) (*P *= 0.009 using the KW test).

The second reason for considering pyruvate kinase as the central node is based on network analysis. Until now, several measures for characterizing node centrality have been proposed. Some famous examples are the utilization of degree centrality, closeness centrality, and betweenness centrality (see Methods for details). Using these centrality measures, we found that pyruvate kinase shows high centrality: it has the second largest degree centrality, the seventh largest closeness centrality, and the fourth largest betweenness centrality (see Additional file 1). Although the enzyme with the highest centrality is a multifunctional one encoded by the gene b1850, we selected pyruvate kinase as the center because of its high visibility. The difference in selection between these enzymes does not influence the shortest path analysis because these enzymes are adjacent to each other on the metabolic network. Similarly, we may observe almost similar results even when other enzymes with high centrality are used as the center, because these enzymes are also distributed near pyruvate kinase (see Additional file 1). In addition, distance distribution can be calculated using metabolic network data (Additional file 1), if other metabolites are selected as the center. Figure 1 shows that the median (or mean) of distance from the center (i.e., pyruvate kinase) slightly increases with chaperonin substrate class in the case of both Kerner's classification and Fujiwara's classification. This result implies that as chaperonin requirement increases, substrate enzymes are more laterally distributed in the metabolic network. However, the frequency distribution of the distance for all metabolic enzymes is almost similar to that for chaperonin-dependent enzymes (i.e., Class III or IV), although the average for Class III or IV substrates (3.65 for Class III) is slightly larger than that for all enzymes (3.58) (*P *= 0.77, according to Student's *t*-test). Therefore, it seems to make sense that Class III or IV enzymes are neutrally located in the metabolic network, and that the chaperonin-independent enzymes are present around the center of the network.

### Increase in distance between substrate enzymes with chaperonin requirement

We next investigated the shortest path length between chaperonin substrate enzymes belonging to the same substrate class as another metric for characterizing the distribution of chaperonin substrates in the metabolic networks. This feature is characterized by the proportion of substrate enzyme pairs separated by the shortest path length *h*, and it is defined as follows: .

**Figure 2:**
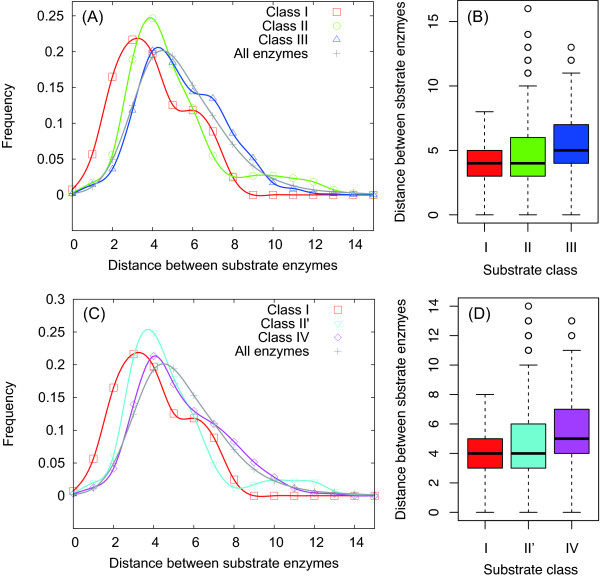
**Distance between enzymes in the same chaperonin substrate class**. The frequency distributions of the distance (i.e., shortest path length) according to Kerner's classification (A) and Fujiwara's classification (C). The boxplots of the distance according to Kerner's classification (B) (*P <*2.2 *× *10^-16 ^using the Kruskal-Wallis (KW) test) and Fujiwara's classification (D) (*P <*2.2 *× *10^-16 ^using the KW test).

As shown in Figure 2, in the case of both Kerner's classification and Fujiwara's classification, the median (or mean) distance between substrate enzymes in the same class slightly increases with the chaperonin substrate class. This result suggests that as chaperonin requirement increases, substrate enzymes in the same class are more discretely located in the metabolic network. Again, the average distance for the chaperonin-dependent enzymes (i.e., Class III or IV) (5.43 for Class III) is similar to that for all metabolic enzymes (5.43) (*P *= 0.95 using the Student's *t*-test), implying the neutral distribution of chaperonin substrates in the metabolic network.

### Traditional network measures can hardly distinguish the differences among the chaperonin substrate classes

Nodal properties, such as the clustering coefficient and centrality measures, obtained from network structures are useful and have been widely utilized for biological networks because they (especially, centrality measures) are correlated with actual bimolecular properties such as the evolutionary rates of proteins [25] or genes [26] and protein essentiality [27]. Thus, on the basis of these previous works, it is also necessary to evaluate whether there are significant differences in the traditional network measures for each node (i.e., enzyme) obtained from the metabolic network structure among the chaperonin substrate classes, which are a bimolecular property.

We focused on 3 well-known centrality measures and clustering coefficients (see Methods for details) and evaluated the network measures of individual enzymes among the chaperonin substrate classes.

**Figure 3:**
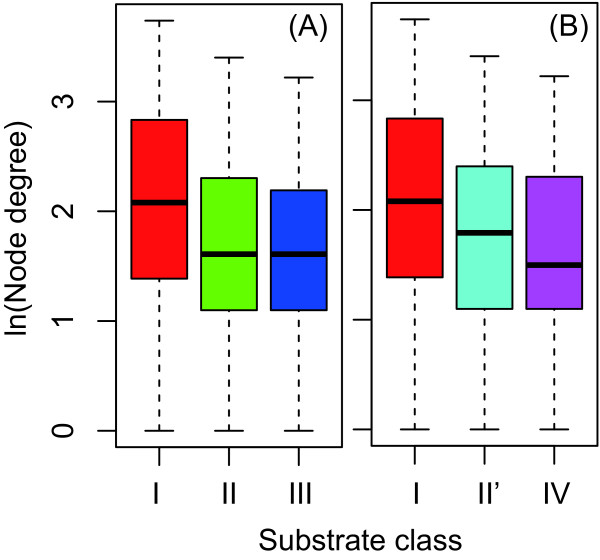
**Difference of node degree (i.e., degree centrality) among the chaperonin substrate classes**. (A) The case of Kerner's classification (*P *= 0.15 using the Kruskal-Wallis (KW) test). (B) The case of Fujiwara's classification (*P *= 0.15 using the KW test). Note that the node degree (i.e., degree centrality) is shown as logarithmic.

As a representation, we show the differences in the node degree (i.e., degree centrality) for each enzyme among the chaperonin substrate classes (Figure 3). The results showed that there were no significant differences (*P *= 0.15 using the Kruskal-Wallis test) although the median of the node degree seems to decrease with increasing the chaperonin substrate classes (e.g., I *>*II (II') ≥ III (IV)).

Similarly, we investigated the differences in other network measures (i.e., the closeness centrality, betweenness centrality, and clustering coefficient) for each enzyme among the chaperonin substrate classes in both cases according to the Kerner's classification and Fujiwara's classification. We defined the significance of the difference of each network measure among the chaperonin substrate classes as the *P *-value obtained from the Kruskal-Wallis test, and showed the difference among the classes using only the *P *-value in order to avoid many redundant figures. If you want figures that show the difference of these network measure among the chaperonin substrate classes, you can obtain such figures using the Additional file 1. The *P *-value is summarized in Table 1.

**Table 1:** Statistical significance of differences in traditional network measures among chaperonin substrate classes

Substrate classification	Closeness centrality	Betweenness centrality	Clustering coefficient
Kerner's	0.05	0.97	0.97
Fujiwara's	0.09	0.66	0.60

The numerals denote the *P *-values obtained from the Kruskal-Wallis test.

Table 1 shows that these traditional network measures showed no significant difference among the chaperonin substrate classes. This result indicates that the traditional network measures hardly distinguish the difference among chaperonin substrate classes. However, we found that the closeness centrality was slightly different among the chaperonin substrate classes (*P <*0.1), and this may be because it is based on the shortest path length.

### Ambiguous difference in the distribution of substrate enzymes in the metabolic network between Class III^- ^and IV

Class III*^- ^*substrates are a subset of Class III substrates, and they are soluble in chaperonin-GroE-depleted cells although they interact with chaperonin. Thus, it is important to determine the difference between Class III*^- ^*and IV, which is related to the differences according to Kerner's classification and Fujiwara's classification. A previous work [22] reported differences in protein features such as the proportion of positively charged residues and hydrophobicity between Class III*^- ^*and IV substrates.

However, we could not determine any clear difference between Class III^- ^and IV substrates in case of both, distance from the center (*P *= 0.48 using the Wilcoxon test) and distance between substrate enzymes belonging to the same chaperonin substrate class (*P *= 0.07 using the Wilcoxon test). However, we concluded that the difference between Class III^- ^and IV substrates is ambiguous because the metabolic network has only 9 Class III^- ^substrate enzymes.

### Novel insight provided by the different distribution patterns of chaperonin substrate enzymes: Comparison with previous works

As shown in the previous sections, we found that the distribution pattern of substrate enzymes differed with respect to chaperonin requirement. Since the previous works showed the striking properties of chaperonin substrates based on the characteristics of individual proteins, our finding provides a novel insight into chaperonin substrate properties because it is based on the relationship with metabolic networks.

Until now, several works [16,20,22,28] have focused on individual protein features in order to identify the striking properties of chaperonin substrates: molecular weight, hydrophobicity, the proportion of charged residues, structural class (i.e., SCOP: Structural Classification of Proteins [29]), and the nucleotide (or amino acid) substitution rate.

**Figure 4:**
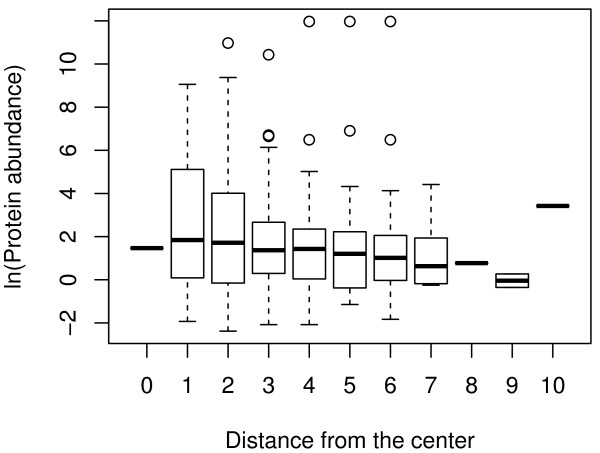
**Relationship between protein abundance and the distance from the center**. There is no difference in protein abundance due to the distance from the center of the metabolic network (*P *= 0.67 using the Kruskal-Wallis test). Note that protein abundance is shown as logarithmic.

Especially, protein abundance may be a prominent example. Kerner *et al*. showed that protein abundance is critically different between chaperonin substrate classes (e.g., see Figure 4B in [20]). Thus, there is a possibility that the distribution pattern of substrate enzymes is caused by a difference in protein abundance. To test this possibility, we investigated the relationship between protein abundance [30] and distance from the center in the metabolic network (see Method for details). We found no correlation between these 2 factors (Figure 4), indicating that protein abundance does not explain the distribution pattern of substrate enzymes in the metabolic network.

Thus, other explanations for the distribution pattern of substrate enzymes are required. We therefore hypothesized that these nontrivial distribution patterns may be explained by evolutionary factors because chaperones, including the chaperonin GroEL, have been suggested to be deeply related to evolution [31,32] (discuss later for details).

### Species specificity of substrate enzymes according to chaperonin requirement

It is also important to investigate chaperonin substrate enzymes from an evolutionary viewpoint. We have focused on the degree of conservation of substrate enzymes among wide-ranging living organisms (see Methods for definition).

**Figure 5:**
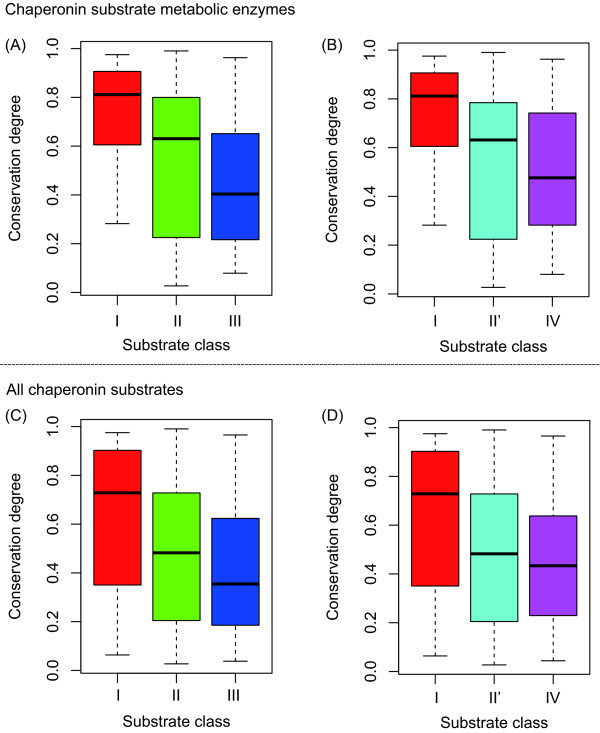
**Degree of conservation of chaperonin substrates**. The difference in the degree of conservation of substrate enzymes among chaperonin substrate classes, according to Kerner's classification (A) (*P *= 0.0008 using the Kruskal-Wallis (KW) test) and Fujiwara's classification (B) (*P *= 0.004 using the KW test). The difference in the degree of conservation for all chaperonin substrates among substrate classes, according to Kerner's classification (C) (*P *= 0.0002 using the KW test) and Fujiwara's classification (D) (*P *= 0.002 using the KW test).

Figure 5 shows that the degree of conservation of chaperonin substrates decreases with the chaperonin substrate class. This result suggests that substrate enzymes are more species-specific, as chaperonin requirement increases. This tendency of the degree of conservation was similar between the substrate enzymes and all chaperonin substrates. Thus, the decrease in the degree of conservation with chaperonin requirement may be a basic property of chaperonin substrates.

The degree of conservation is believed to be related with the evolutionary age because it is expected that well-conserved genes emerged in early evolution. For example, pyruvate kinase and enolase, which are involved in glycolysis and/or gluconeogenesis, are well conserved among a wide range of living organisms, suggesting that these metabolic pathways are ancestral [33,34]. Therefore, we can explain the lower degree of conservation by the emergence of chaperonin-dependent substrates later on in evolutionary history. Note that it is not necessary that enzymes that are orthologs of chaperonin-dependent substrates in *E. coli *require GroE for protein folding. For example, in the case of *Ureaplasma urealyticum*, which has no chaperonin, it has been confirmed that several orthologs of chaperonin substrates (Class IV in this case) show no chaperonin requirement [22].

The distribution pattern of chaperonin substrate enzymes in the metabolic network further implies that *U. urealyticum *has no chaperonin. Since some Mollicutes, including *U. urealyticum*, have no GroEL [35], it is important to investigate their adaptation to the lack of GroEL. *U. urealyticum *is a mucosal pathogen. In *U. urealyticum*, except for the central metabolic pathway, many other metabolic pathways are dependent on the metabolism of the host species [36]. As shown in the previous section, few chaperonin-dependent enzymes are located at the center of the metabolic network. Thus, it is possible that *U. urealyticum *metabolism can take place in the absence of chaperonin. Although this is just a speculation, it may provide a clue about the survival potential of species in the absence of chaperonins.

### Hypothesis for the expansion of metabolic networks involving chaperonin

In this study, we demonstrate 2 main results: according to the chaperonin requirement, (i) substrate enzymes are more clustered away from the center of metabolic networks, and (ii) they may have been incorporated later into the metabolic network in evolutionary history. These results suggest that the expansion of metabolic networks is due to chaperonin. This suggestion is inspired by the proposal by Rutherford and Lindquist [31], in which the authors conclude that chaperones can accelerate phenotypic diversity (i.e., evolution). In general, since phenotypes are related to metabolism, we speculated that the chaperonin GroE mediates metabolic network evolution. This network expansion hypothesis may be able to explain the relationship between the position of substrate enzymes in the metabolic networks and the chaperonin requirement as follows.

Ancestral metabolic networks may have been smaller, and its enzymes may have functioned independently of chaperonins. However, the emergence of chaperonins may have induced enzymatic diversity (i.e., increased types of metabolic enzymes), and resulted in the expansion of the metabolic network. Several previous works support this notion. Tokuriki and Tawfik [32] reported the modification of enzymatic specificity (i.e., change in enzymatic function) induced by the overexpression of GroEL through experimental evolution. Protein mutations may have been selected with relative ease because chaperonins assisted in the formation of naive structures, and subsequently led to accelerative changes in proteins. In fact, the nucleotide (or amino acid) substitution rate of chaperonin-dependent proteins is faster than that of other enzymes [37,38]. Moreover, several previous works have stated that metabolic network evolution is due to the modification of enzymatic specificity, and this was confirmed in several biosynthetic pathways, such as the citrate cycle and lysine biosynthetic pathway (e.g., reviewed in [39]), which possess chaperonin-dependent substrate enzymes.

For the above-mentioned reasons, we believe that the increase in enzyme diversity induced by chaperonins caused the expansion of metabolic networks. Through this expansion process, as a result, chaperonin-dependent enzymes (i.e., Class III or IV) might evolve to be distributed at the side of the metabolic network.

In addition, note that the absence of differences in the distributions of chaperonin-dependent enzymes and all other enzymes, as shown in Figures 1 and 2, does not contradict the idea of network expansion due to chaperonin. Seemingly, the absence of differences may imply that the chaperonin-dependent enzymes are naturally distributed and not clustered at the side of the network. However, this distribution tendency is because of the small-world property of networks [23,40], which indicates that the shortest path length *h *increases approximately with the logarithmic order of the network size *N *(i.e., the number of nodes): *h *∝ ln *N*. Considering the small-world property, the distance (shortest path length) undergoes very little change for a large network. The chaperonin-dependent enzymes may have emerged after the network partially expanded. This means that the network size was already relatively large. Therefore, the distance distribution of chaperonin-dependent enzymes is almost similar to that of all metabolic enzymes.

The small-world property suggests that the distance distribution of early-emerged enzymes (i.e., Class I and II) rather than late-emerged enzymes (i.e., Class III and IV) is different from that of all enzymes because the Class I and II substrates may occur in the relatively small network. Because this distribution tendency is observed in Figures 1 and 2, we concluded that the distribution pattern of substrate enzymes indicates the metabolic network expansion due to chaperonin.

According to the hypothesis for the expansion of metabolic networks due to the chaperonin, the difference in chaperonin-dependent substrates among living organisms is because the substrates might have been recently acquired (or because they are species-specific). Since comprehensive analysis of chaperonin-dependent substrates among many species has still not been completed, we could not evaluate this prediction. However, the chaperonin (GroE) substrates from *E. coli *are different from those from the thermophilic bacterium *Thermus thermophilus *[41], the gram-positive bacterium *Bacillus subtilis *[42], and the archaeon *Mathanosarcina mazei *[43]. These results may support this hypothesis.

## Conclusions

We investigated the distribution of chaperonin substrate enzymes on the *E. coli *metabolic network, and revealed the relationship between metabolism and chaperonins in more detail. In particular, network analysis showed that the substrate enzymes are more laterally distributed in the network with increase in chaperonin requirement. In addition, it was suggested that chaperonin-dependent enzymes were acquired later on in evolutionary history. These results imply the expansion of metabolic networks due to chaperonins; thus, they provide an example for the existing hypothesis on chaperonin-induced diversity (or evolution). This finding may provide new insights into the evolution of the metabolic network evolution and the roles of chaperonins in living systems.

## Materials and methods

### Construction of the *E. coli *metabolic network

**Figure 6:**
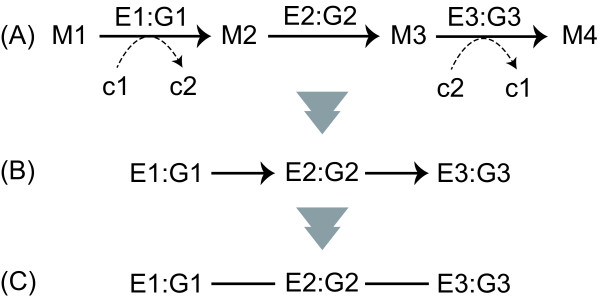
**Schematic diagram of the construction of metabolic reaction networks**. (A) A general representation of the metabolic pathway. Reactions are represented as enzymes (E1, E2, and E3) and corresponding genes (G1, G2, and G3). For example, E1:G1 means that the gene G1 encodes enzyme E1. M1, M2, and M3 correspond to metabolic compounds. C1, c2, and c3 indicate currency metabolites such as ATP and NADH. (B) The metabolic reaction network obtained from (A). (C) The metabolic reaction network is finally represented as an undirected network because of the shortest path analysis.

We downloaded the XML files (version 0.7.1) storing the metabolic network of *E. coli *K-12 MG1655 from the KEGG (Kyoto Encyclopedia of Genes and Genomes) database [44]http://www.genome.jp/kegg-bin/show_organism?org=eco. Based on the XML data, we constructed the metabolic reaction networks [6,7,23,25], in which the nodes and edges are metabolic enzymes (reactions) and interjacent chemical compounds, respectively. In particular, the reaction network was obtained as follows. As an example, we consider a metabolic pathway that consists of 3 metabolic enzymes, E1, E2, and E3, whose corresponding genes are G1, G2, and G3, respectively (Figure 6A). Basically, an edge is drawn between 2 reactions (nodes) if at least 1 product of a reaction corresponds to at least 1 substrate of the other reaction. For example, the link E1:G1*→*E2:G2 is obtained because M2 acts as both the product of E1 and the substrate of E2. In this case, however, the currency metabolites, such as water, ATP, and NADH, generate links without essential biological roles. For example, we consider the reactions E1:G1 and E3:G3, whose interjacent chemical compound is the currency metabolite c2 (e.g., ATP). When considering metabolic reaction steps, it is not appropriate to draw an edge from E1:G1 to E3:G3 because currency metabolites play simple roles, such as energy exchange, exchange of a proton, or phosphate moiety; thus, the metabolic reaction network should be represented as in Figure 6B. Considering this situation, many researchers omit currency metabolites when constructing biologically appropriate reaction networks [6,7,23,25]. However, these currency metabolites do play a role in metabolic networks and, therefore, should not be removed.

To reduce the effect of the above problem as much as possible, we used the XML files from the KEGG database (KGML files) in which the metabolic reactions described consist of essential substrate-product pairs (represented as solid arrows in Figure 6A) manually curated based on the information available in the literature (but partially obtained using the automatic systems [45] and inspired by atomic mapping [3]). The biologically unsuitable links mentioned above were excluded by using only the essential substrate-product pairs in the KGML files where edges between reactions (nodes) are drawn (see also Figure 6B).

The distribution of chaperonin substrates in the metabolic network is characterized on the basis of the shortest path length. Because of this, the existence of unreachable node pairs produces an unsuitable result. For example, the frequency of the shortest path length between a node pair may be overestimated when a network has unreachable node pairs.

To obtain reachable node pairs, the largest strongly connected component extracted from a directed network may be considered. However, the strongly connected component may not be suitable for comprehensive network analysis because its size (i.e., the number of nodes) may be too small. To obtain as many reachable node pairs as possible, we finally focused on the largest connected component extracted from an undirected network (i.e., the largest weakly connected component represented as an undirected network). In particular, we performed the following procedure. (1) We represented the metabolic network, which is expressed as a directed network (Figure 6B), as an undirected network (Figure 6C). (2) We extracted the largest connected component from this undirected network.

Through this procedure, this metabolic reaction network is expressed as an undirected (and unweighted) network in which the paths between all node pairs are possible. We finally obtained metabolic reaction networks consisting of 615 nodes and 2,083 undirected edges. A comprehensive shortest path analysis is possible by using this network because the largest connected component covers most of the original metabolic networks (the number of nodes in the largest connected component and in the original network were 615 and 624, respectively) although it has a limitation that the edge direction is not considered.

### Centrality measures

The node degree is the simplest measure of centrality, and it is defined as the number of neighbors of a node. This centrality (called degree centrality) assumes that high-degree nodes show high centrality.

The closeness centrality [46] is based on the shortest path length between nodes *i *and *j*, *d*(*i, j*). When the average path length between a node and the other nodes is relatively short, the centrality of such a node may be high. On the basis of this interpretation, the centrality of node *i *is expressed as .

If a walker moves from one node to another node via the shortest path, then the nodes with a large number of visits by the walker may have high centrality. The betweenness centrality of node *i *is defined as [46], where *σ_st_*(*i*) and *σ_st _*are the number of shortest paths between nodes *s *and *t*, on which there is node *i*, and the number of shortest paths between nodes *s *and *t*, respectively. For normalization, the betweenness centrality is finally divided by the maximum value.

### Clustering coefficient

The clustering coefficient of node *i *characterizes the edge density among neighbors of node *i*, and it is defined as 2*M_i_/*[*k_i_*(*k_i _- *1)] [40,47], where *M_i _*is the number of edges drawn among neighbors of node *i*, and *k_i _*is the number of neighbors of node *i*. [*k_i_*(*k_i _- *1)]/2 indicates the maximum number of possible edges that can be drawn among *k_i _*neighbors.

### Protein abundance

In Figure 4, protein abundance data is shown by the exponentially modified protein abundance indices (emPAIs) that are available in the Additional File two of [30]. We evaluated the relationship between the distance from the center and protein abundance for 409 proteins (approximately 50% of the genes in the metabolic network).

### Degree of conservation of chaperonin substrates

The degree of conservation is calculated based on the KEGG orthology (KO) database [44]. The KO database stores the list of orthologous genes (available at http://www.genome.jp/kegg/ko.html); thus, it is similar to the Clusters of Orthologous Group (COG) database [48]. However, we selected the KO database because it is applicable to more living organisms than the COG database.

The degree of conservation is simply defined as *S_i_/S*_total_, where *S_i _*corresponds to the number of species possessing at least 1 orthologous gene for the gene *i *coding the chaperonin substrate. *S*_total _denotes the total number of species that are available in the KO database, which is 1,368 (as of 19 January 2011).

## Competing interests

The authors declare that they have no competing interests.

## Authors' contributions

KT designed this research, analyzed the data, and drafted the manuscript. TN and HT provided suggestions for data analysis, and they discussed the results with KT. All authors read and approved of the final manuscript.

## Supplementary Material

Additional file 1 **Description for each enzyme node and metabolic network data**. This Excel file contains 2 sheets. One sheet includes the gene identifiers (b-numbers), the chaperonin substrate class, and the network measures, for each enzyme node. The other sheet includes the edge list (i.e., the binary relationship between source nodes and target nodes) for the metabolic network of *Escherichia coli*. Click here for file
